# Dr. Wm. E. Marshall

**Published:** 1918-02

**Authors:** 


**Dr. Wm. E. Marshall,** (college and date to be supplied in proof), died Jan. 6 at Cobourg, Ont. He was formerly a resident of Buffalo.

---

The Jour. of the A. M. A. noted the deaths of 2,300 physicians in the U. S. and Canada during 1917, estimated at 14.37:1000. The average annual mortality of 1902-1917 was 15.53:1000. Deaths occurred at all ages from 21 to 100, the maximum for any one age being 63 at 61. The average age at death was 59¾, for the whole period, 60¾ for 1917. The average period of practice was 31 years 2 1/3 months for the whole period, 32 years 7 months for 1917. The principal causes of death were: senility 388, heart disease 202, cerebral haemorrhage 195, pneumonia 189, accidents 111, nephritis 86, surgical operation 66, tuberculosis 44 (less than 2% of the total), suicide 31. Homicide caused 16 deaths. Of the accidental deaths, 41 were due directly to automobiles and 9 to automobiles struck by trains at crossings. It will be noted that the present editor's estimate that 2% of all physicians died from automobile accidents was exceeded.

